# How Perceived Sensory Dimensions of Forest Park Are Associated with Stress Restoration in Beijing?

**DOI:** 10.3390/ijerph19020883

**Published:** 2022-01-13

**Authors:** Congying An, Jinglan Liu, Qiaohui Liu, Yuqi Liu, Xiaoli Fan, Yishen Hu

**Affiliations:** 1 School of Ecology and Nature Conservation, Beijing Forestry University, Beijing 100083, China; acy19961219@163.com (C.A.); rachelyuqi@gmail.com (Y.L.); fanxiao0802@163.com (X.F.); hys16637102261@163.com (Y.H.); 2 College of Forestry, Beijing Forestry University, Beijing 100083, China; liuqiaohui202109@163.com

**Keywords:** perceived sensory dimensions (PSDs), stress restoration, forest park, the short-version revised restoration scale (SRRS)

## Abstract

A growing number of studies suggest that the perceived sensory dimensions (PSDs) of green space are associated with stress restoration offered by restorative environment. However, there is little known about PSDs and stress restoration as well as their relationship to forest park. To fill this gap, an on-site questionnaire survey was conducted in three forest parks in Beijing, as a result of which a total number of 432 completed responses were collected and analyzed. The mean values of PSDs were used to represent PSDs of forest park. Using independent sample *t*-test and ANOVA, this study analyzed the individual characteristics that affected PSDs and stress restoration. Linear mixed model was used to identify the relationship between PSDs and stress restoration of forest park, which took into account the interactions of stress level and PSDs. The results showed that: (1) the perceived degree of PSDs in forest park from strong to weak was Serene, Space, Nature, Rich in species, Prospect, Refuge, Social and Culture, which varied with visitors’ gender, age, level of stress, visit frequency, activity intensity, visit duration and commuting time; (2) in PSDs, Refuge, Serene, Social and Prospect had significantly positive effects on the stress restoration of forest parks (3) there was no significant difference in the effect of the eight PSDs on the stress restoration between different stress groups; (4) stress restoration was influenced by visitors’ gender, age, visit frequency and visit duration. These findings can offer references for managers to improve the health benefits of forest park for visitors, and can enrich the knowledge about PSDs and stress restoration.

## 1. Introduction

As urbanization continues to expand and deepen, people have less and less access to nature, and face higher-than-normal levels of stress, which can lead to physiological illnesses such as heart disease and diabetes, as well as many psychological illnesses [1,2,3]. Studies have found that green spaces can significantly reduce the level of stress and promote physical and mental recovery [4,5,6,7,8], and also can improve people’s health condition [9,10,11]. The environment that can provide people with restorative experience is called a restorative environment [12]. Many studies focused on environmental characteristics that influence the restoration potential, such as landscape location and vegetation density [13], landscape type [14], forest stand structure [15], visual preference [16] and perceived features [17]. Among them, it is particularly important to evaluate the characteristics of restorative environment from the perspective of people’s perception [18].

### 1.1. Relevant Theories

Two complementary theories, attention restoration theory (ART) and psycho-evolutionary theory (PET) (also known as stress reduction theory) provided theoretical reference for researches on both perceived characteristics and restorative potential of environment. When people are in a restorative environment, attention recovery processes and stress reduction processes interact thereby producing cognitive and emotional benefits [19].

ART divides attention into involuntary attention and voluntary attention (also known as directed attention) [20]. According to ART, excessive concentration can lead to “directed attention fatigue”, while natural environment has “soft fascination” [10], so being immersed in nature appeals to people’s preferences for a feeling of relaxing, thus improves attention recovery and cognitive performance [20]. ART posits that restorative environment needs be equipped with *Fascination*, *Being away*, *Extent* and *Compatibility* [20], which are the bases for perceived sensory dimensions (PSDs) to be identified and classified.

PET considers that humans somehow adapt to natural environments rather than urban ones because humans have evolved in natural environments for so long [21]. Moreover, natural environments can activate the parasympathetic nervous system [22]. Therefore, people staying in natural environments would experience stress reduction on both physiological and psychological levels [23], as well as positive changes in emotional states [24].

### 1.2. Perceived Sensory Dimensions (PSDs)

Different types of people in the same environment experience different character-istics, some of which are beneficial to people’s health [25]. Measuring environmental attributes from the perspective of users’ perception can identify features that cannot be identified by objective measurement [26]. Furthermore, features identified from a perceptual perspective are directly related to restoration, helping researchers determine which environmental features are popular or restorative. There is a connection between people’s perception of the surroundings through their senses and people’s health [17]. PSDs were developed to evaluate the characteristics of restorative environment from the perception, which consists of eight dimensions: Nature (wild nature not created by humans), Culture (an environment containing an essence of human culture), Prospect (the area with an open view), Social (an environment that is equipped for social activities), Space (a green environment that is spacious and free and has a certain amount of connectedness), Rich in species (an environment which offers a variety of animals and plants), Refuge (a hidden and safe environment, where people can watch other people being active), Serene (a peaceful and secure place) [17,22]. Specially, PSDs correspond to people’s needs for rest, exercise, social contact, entertainment and safety, so it can identify environmental characteristics that reduce pressure and promote human recovery [27], thus offering direction for constructing environment with good stress restoration. PSDs had been recognized as an effective tool to qualitatively analyze and evaluate the perceived characteristics of restorative environment, helping to measure the quality of green space for restorative experiences [24,28]. They also have good application effects in the cultural and environmental background of China [29,30].

The relationship between PSDs and stress restoration offered by a restorative experience of environment has always been a hot topic. In the questionnaire research, urban parks with the stress restoration needed to have Nature, Rich in species, Serene and Refuge [31], while for small public urban green space (SPUGS), Social and Serene were necessary [28]. The result of the laboratory experiment suggested that all the eight PSDs had a significant impact on the stress restoration, and Nature and Serene were the most important factors [27]. Serene, Refuge, and Nature had been proved to be associated with stress restoration for college students [22,32]. Similarly, in the study of adolescents, Nature, Refuge, and Prospect were found to have stress restoration effect [33]. For patients suffering from stress-related diseases, Refuge, Serene, Nature, and Rich in species of gardens can affect the stress restoration [34]. It can be seen that if the research methods, environment types and subjects are different, the results will be different for PSDs research. Further, more studies are needed to analyze the relationship between PSDs and stress restoration. Moreover, in the context of Chinese culture and environmental background, it is not clear how the PSDs are related to the stress restoration.

### 1.3. Studies on Forest Park

In Beijing, forest park, a natural place where people often go to relax, is the core of providing “forest health” service for people [35]. Forest environments including forest park are the most concerned restorative environments. Researchers have documented the effects of forest environments on stress and anxiety reduction, as well as mood improvement [36,37,38]. At present, the researchers concentrate on not only features of the forest being of restorative potential, but also mechanisms and other factors affecting the restorative potential. On the basis of studies, preference, place attachment and appropriate physical activity were helpful to restoration [39,40,41,42]. Equally important, demographic and access characteristics also influenced the recovery benefits that people derived from forest environments [43,44]. However, to date, studies have rarely investigated the influencing factors both of PSDs and stress restoration, as well as their relationship in forest park.

### 1.4. Study Goals

Compared with the types of urban green spaces studied previously, such as small public urban green spaces, urban park and garden, forest park has characteristics of large area, richness of species diversity, few visitors and less infrastructure. Therefore, we hypothesize that the PSDs and stress restoration and their relation to forest park is different from other types of urban green spaces. The objective of this study was to explore how PSDs of forest park are associated with stress restoration in Beijing. The specific research contents are identifying:

(I) PSDs of forest park and their influencing factors;
(II) the relationship between PSDs and stress restoration in forest park;
(III) individual characteristics affecting stress restoration in forest park.

## 2. Materials and Methods

### 2.1. Study Sites

**Figure 1:**
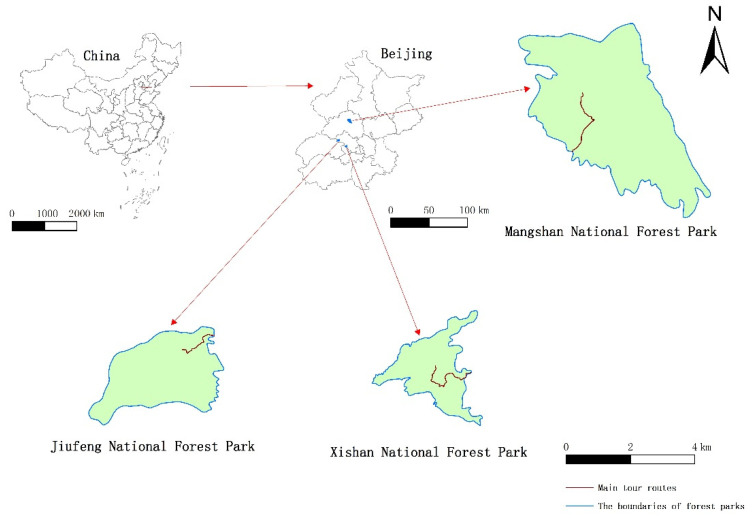
The map of research sites and main tour routes in each forest park.

**Figure 2:**
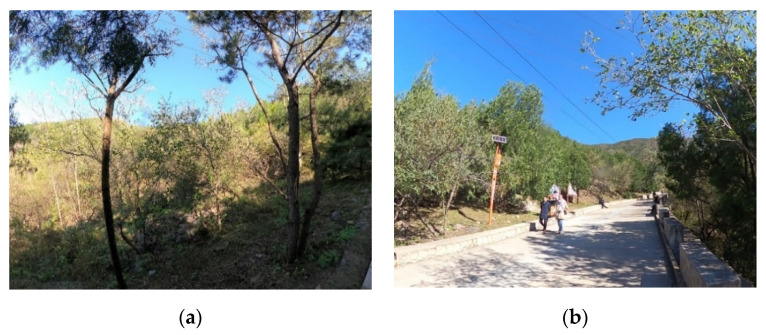
Forest landscape and artificial facilities in Mangshan National Forest Park (MS): (**a**) Forest landscape; (**b**) Artificial facilities.

**Figure 3:**
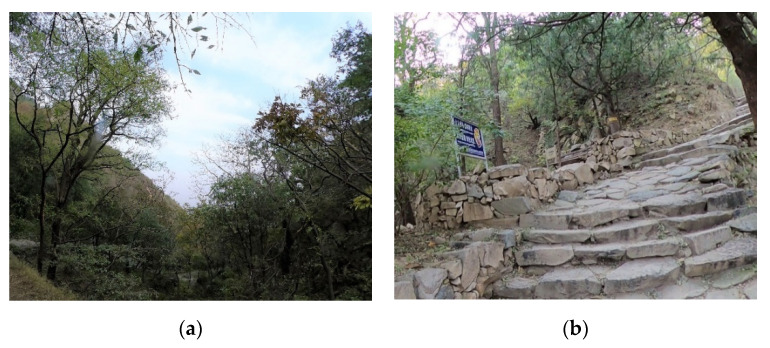
Forest landscape and artificial facilities in Jiufeng National Forest Park (JF): (**a**) Forest landscape; (**b**) Artificial facilities.

**Figure 4:**
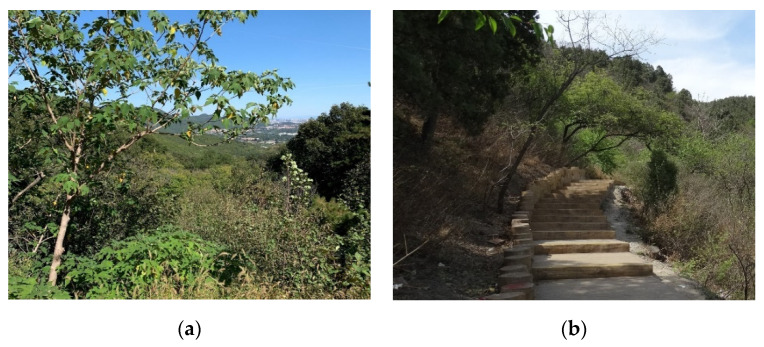
Forest landscape and artificial facilities in Xishan National Forest Park (XS): (**a**) Forest landscape; (**b**) Artificial facilities.

The research was conducted in Mangshan National Forest Park (MS), Jiufeng National Forest Park (JF) and Xishan National Forest Park (XS), which are located in the suburb of Beijing, the capital of China (Figure 1). Mangshan National Forest Park covers an area of about 1760 ha, about 35 km away from the city center. Jiufeng National Forest Park covers an area of about 729 ha, about 25 km away from the city center. Xishan National Forest Park covers an area of about 442 ha and is about 15 km away from the city center. The three forest parks are dominated by forest landscape resources and have a certain number of artificial facilities (Figure 2, Figure 3 and Figure 4).

### 2.2. Measuring Tool

We designed the questionnaire by referring to relevant literatures [28,29,30]. The questionnaire was divided into three parts.

The first part was the individual characteristics of visitors, including gender, age, preference for forest park for outdoor activity or not (Prefer), level of stress in the last month (LS) and visiting characteristics of forest park (VCs). Six questions were set for visiting characteristics of forest park: visit purpose (VP), visit frequency (VF), activity intensity (AI), visit duration (VD), commuting time (CT) and number of companions (NC). We determined LS of the visitors by directly asking them about their level of stress in the last month. The options were divided into five levels. We defined the visitors who chose “3” and “4” as the stressed visitors (SV), and others as the average visitors (AV) [45]. Regarding visiting characteristics of forest park, concrete items can be seen in Table 1.

The second part was PSDs scale [17], which deleted the activities prohibited in Beijing’s forest parks, such as lighting fires, playing football and so on. At the same time, the selection of variables referred to the application of PSDs by previous research [22]. Consequently, according to the actual conditions and previous applications, we adopt a total of 28 variables of PSDs (Table 2).

The third part was SRRS, a reliable and valid self-rating measure of the restorative potential of natural environments [46]. The scale consists of eight variables, which are divided into four dimensions of emotional response, physiological response, cognitive response and behavioral response [46]. Compared with the Stress Restoration Scale (PRS) which was more widely used in stress restoration, SRRS adopts a broader concept of recovery [22], and it is much more concise [23]. Therefore, SRRS was used in this research to measure the stress restoration of forest park.

All items in the second part and the third part in the questionnaire had a 5-point Likert scale ranging from 1 = completely disagree to 5 = completely agree.

### 2.3. Data Collection

Questionnaire survey was conducted on site in three forest parks, respectively, on 6, 13 and 20 June 2021 under similar weather conditions. The questionnaires were distributed to visitors who had already visited the forest park and through the main tour routes (Figure 1). At first, visitors were informed of the purpose and content of the survey, then willing participants were invited to fill out questionnaires during their stay in the area so that the answers would reflect their immediate experience. Participants were selected among visitors in each forest park regardless of their social-demographic characteristics or educational background.

### 2.4. Data Analysis

In this study, we used Excel 2010 (Microsoft Corporation, Seattle, WA, USA) to record raw data from all questionnaires. All statistical analyses were processed by SPSS 26.0 (IBM, Armonk, NY, USA).

First of all, since the PSDs proposed by Grahn et al. [17] were somewhat deleted in this study, reliability and validity test of the scale were conducted. At the same time, in the past application of SRRS, as researchers mainly used slides as experimental materials to study the stress restoration of environment [47,48,49], only Liu Qunyue applied this scale in the real natural environment in his research on the relationship between the naturalness of university green space and its stress restoration [23]. Therefore, this study verified the application effect of the SRRS in forest park.

Then, we used the mean value of each dimension as PSDs of forest park. Independent sample *t*-test and one-way analysis of variance (ANOVA) were used to determine the individual characteristics that affected the PSDs of forest park. Gender, Prefer and LS were dichotomous variables, so independent sample *t*-test was used. Age and visiting characteristics to forest park were multi-classification variables, so ANOVA was used.

To determine the impacts of PSDs of forest park on stress restoration, we used a linear mixed model. In order to control the possible differences between three forest parks, we took “forest park name” as a random factor and eight PSDs as fixed factors. According to the calculation method of SRRS by other researchers [22,50], we first reverse processed the two variables of physiological response, then calculated the average score of eight variables to obtain the stress restoration index, which was taken as the dependent variable. In particular, considering the studies which argued that stress level had influence on the perception of environment characteristics [45,51], the interactions of LS and the eight PSDs were incorporated into the model for secondary analysis. We analyzed the significance of the interactions and compared the model fitting effects before and after including the interactions, in order to determine whether the interactions of the LS and the eight PSDs affect the stress restoration of forest park. In addition, as individual characteristics may have impacts on the stress restoration [33], we took the stress restoration index as the dependent variable, and also used independent sample *t*-test for dichotomous variables and ANOVA for multi-classification variables to explore the individual characteristics that affect the stress restoration index. After that, least significant difference (LSD) was used to do a post hoc test to accurately analyze the specific impacts of individual characteristics on the stress restoration.

## 3. Results

### 3.1. Individual Characteristics of the Respondents

A total of 490 questionnaires were collected, of which 432 were valid, with an effective rate of 88%. The questionnaires issued in each forest park are shown in Table 3.

First, we described the gender, age, Prefer and LS of respondents, and the specific results are shown in the Table 4. Among 432 valid questionnaires, there were slightly more men than women, and the gender ratio was evenly distributed in the three forest parks. In terms of age, the majority of them were people aged 26–40 in all three forest parks, followed by people aged over 40 years old, and then people aged 18–25; 70.6% of the respondents preferred forest park as outdoor activity site, and 44.4% of the respondents chose “4” or “5” on LS, who were considered as the stressed visitors, and others were considered as the average visitors.

**Figure 5:**
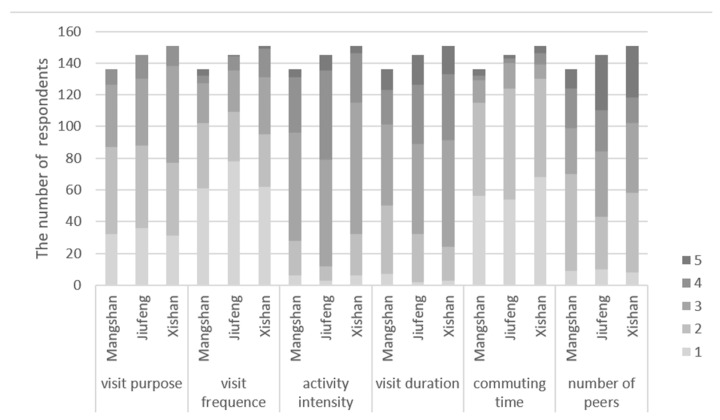
Visiting characteristics to forest park. For visit purpose: 1 = exercising, 2 = relaxing, 3 = being with families or friends, 4 = observing nature. For other visiting characteristics, “1” to “5” means “very few” to “very much” of visit frequency, and “very low” to “very high” of activity intensity, “less than one hour” to “more than four hours” of visit duration and commuting time, “zero” to “four or more” of number of companions.

In terms of visiting characteristics of forest park, there was little difference between the three forest parks (Figure 5). Most respondents visited forest parks for the purposes of “relaxing” and “being with families or friends”. The visit frequency of respondents to the three forest parks was mainly “few”. As for the activity intensity, respondents in JF thought the activity intensity was slightly stronger than MS and XS. The number of respondents with visit duration in “1–2 h” in MS were one of the most; the visit duration of respondents in JF and XS were similar, mainly in “2–3 h”. The commuting time in the three forest parks was similar, mainly in “less than one hour” and “1–2 h”. In number of companions, the number of “one companion” were the most in MS and XS; the number of “two companions” were the most in JF.

### 3.2. The Application Effects of the PSDs and SRRS

The results of PSDs reliability test and validity test showed that the Cronbach α coefficient of the scale was 0.876, indicating that the scale had good internal consistency and high reliability. In terms of validity, the KMO value was 0.873, and the significant value of Bartlett’s sphericity test was 0.000, suggesting that the scale had good validity.

The results of SRRS reliability test and validity test showed that the Cronbach α coefficient of the scale was 0.802, indicating that the scale had good internal consistency and high reliability. About validity, the KMO value of the scale was 0.745, and the significant value of Bartlett’s sphericity test was 0.000, indicating that the scale had good validity. Four common factors were obtained after factor analysis of SRRS, and the factor load matrix obtained after rotation of the common factors was shown in Table 5, which was consistent with the result of Han [46]. Therefore, this scale had good application effect in forest park and can be further studied.

### 3.3. PSDs of Forest Park and Its Influencing Factors

**Figure 6:**
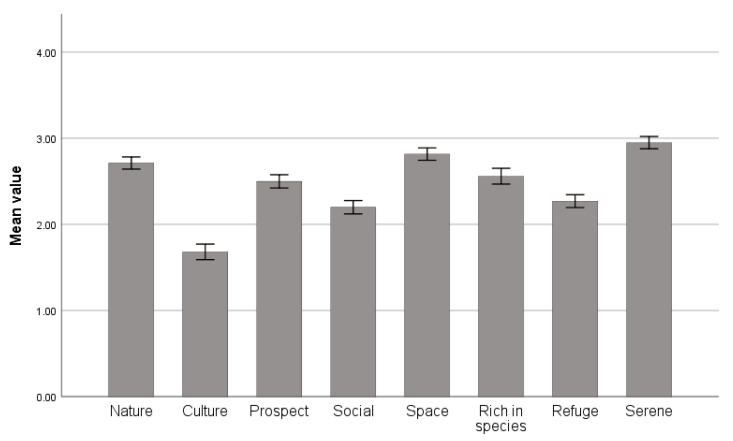
The mean value of each dimension of PSDs. Data are presented as the means ± SEs.

The mean value of each dimension can be seen in Figure 6. The results showed that the perceived degree of PSDs in forest park from strong to weak is Serene, Space, Nature, Rich in species, Prospect, Refuge, Social and Culture.

The independent sample *t*-test results with gender, Prefer and LS as grouped variables and the eight PSDs as test variables were shown in Table 6. The results suggested that visitors’ perception of Nature, Refuge and Serene varied by gender, with women giving significantly higher ratings to the three dimensions than men. Visitors’ perception of each dimension of forest park was not affected by Prefer. Visitors’ perception of the Culture of forest park varies with the LS, and the perception score of the Culture of stressed visitors is significantly lower than the average visitors.

The results of ANOVA with age and visiting characteristics to forest park as independent variables and the PSDs as dependent variables are shown in Table 7. Activity intensity and visit duration had significant effects on the perception of Nature. Prospect was affected by commuting time. Age had an impact on the perception of both Space and Rich in species. Refuge was influenced by visit frequency and visit duration, and visit duration also affected the perception of Serene. Nevertheless, Culture and Social were not affected by Age and visiting characteristics to forest park.

### 3.4. The Relationship between PSDs and Stress Restoration in Forest Park

To test the effect of PSDs on stress restoration of forest park, we constructed a linear mixed model for analysis. First, we performed collinearity test on the model, and the results showed that the variance inflation factor (VIF) between variables were all less than 3, indicating low collinearity between variables. Correlation test showed that variables had low correlation (Pearson correlation, r < 0.6). The results of the model showed that the estimated covariance of random effects was less than 0.001, indicating that the differences of the relationship between PSDs and stress restoration among the three forest parks were very low. AIC of the model was 648.215. Among the eight PSDs, Refuge, Serene, Social and Prospect had significant positive effects on the stress restoration (Table 8). Refuge and Serene had similar and the most significant influence on stress restoration, followed by Social. Prospect had the weakest effect on stress restoration. Nature, Culture, Space and Rich in species had no significant effect on stress restoration.

Then, the interactions between LS and eight PSDs were incorporated into the model for analysis, and the results showed that AIC value increased from 667.858 to 675.227 and none of the interactions were significant. The fitting effect of the model decreased, indicating that the model failed to explain the more variation in the data. Therefore, the interactions between LS and eight PSDs had no significant effect on stress restoration, and it was unreasonable to include them in the model.

### 3.5. Individual Characteristics Affecting Stress Restoration in Forest Park

The results of independent sample *t*-test suggested that the stress restoration varied with gender, and the assessment of women to stress restoration was significantly higher than men (mean difference = 0.110, *p* = 0.046). However, Prefer and LS had no significant effect on the stress restoration.

The results of ANOVA showed that age and visit frequency had significant influence on the stress restoration (Table 9). Further, visitors aged 18–25 had a significantly lower stress restoration than other age groups (Table 10). Those visitors whose visit frequency were “very few” and “few” rated the stress restoration significantly lower than those whose visit frequency were “moderately”, “much” and “very much” (Table 11). Hence, visit frequency was positively correlated with stress restoration. In addition, the post hoc test results suggested that visitors whose visit duration were “2–3 h” (mean difference = 0.158, *p* = 0.03) and “more than 4 h” (mean difference = 0.247, *p* = 0.013) had significantly higher assessment of stress restoration than those whose visit duration were “1–2 h”. ANOVA and post hoc tests were not significant for visit purpose, activity intensity, commuting time and number of companions.

## 4. Discussion

### 4.1. PSDs of Forest Park and Its Influencing Factors

The perceived degree of PSDs in forest park from strong to weak was Serene, Space, Nature, Rich in species, Prospect, Refuge, Social and Culture, which was consistent to some extent with previous result [17]. In addition, Hong Chen et al. [29] evaluated the environment of urban forests in China, and found that the highest score of Nature and Rich in species were obtained, followed by Serene, Space and Refuge, and the lowest score were Prospect, Culture and Social. The reason for the different results may be explained by the type of different environments [29,30,52]. According to our survey, compared with urban forests, forest parks have fewer visitors, higher altitude, larger terrain fluctuations and more open views, so Serene, Space and Prospect are more easily perceived.

In terms of the individual factors influencing the perception of eight PSDs, this study found that gender, age, LS, visit frequency, activity intensity, visit duration and commuting time all affected the perception of visitors in different dimensions. This result verified the previous research [29], that is, individual characteristics and green space using characteristics affect users’ perception of the eight PSDs. On the other hand, a study had concluded that the perception of PSDs by green space users is consistent in gender, age, frequency of visit and activity type [30]. Therefore, whether individual characteristics and the using of green space have impacts on the perception of PSDs needs to be further studied to get more reliable conclusions.

### 4.2. The Effects of PSDs to Stress Restoration

Linear mixed model results displayed that Refuge, Serene, Social and Prospect had significant positive effects on stress restoration in forest park. All four dimensions have been proved to be significantly correlated with stress restoration in previous studies.

Refuge is a preferred feature of the environment that is critical to human survival based on the Prospect-Refuge Theory (PRT) [53]. People with sadness and tension are more willing to be alone in a private and safe place [54]. Refuge satisfies this need of people and thus improves stress restoration. Studies on small public urban green space (SPUGS) have found people’s preference for Refuge [28]. By an interpretative phenomenological analysis (IPA), Stigsdotter et al. [32] also believed that Refuge has an important influence on stress restoration. What is more, forest stand density and canopy density have been verified to be associated with human recovery [13,15,55,56], which also provided evidences for the recovery of Refuge.

Serene is one of the main reasons for people to visit green spaces [17]. As previously noted, Serene had an important effect on stress restoration [26,57,58,59]. Grahn et al. [17] pointed out that Serene was an undisturbed, quiet and calm environment, which means Serene not only refers to the absence of noise, but also requires soothing sounds [57]. On the other hand, noise can cause people to have stress and related diseases [60], which also implied the important role of Serene in human health.

There were different conclusions about the influence of Social on stress restoration. Stressed people preferred the environment with low sociality [17], because they were difficult to understand, sympathize with and tolerate others [61], therefore, Social had a negative impact on stress restoration [22]. However, in small public urban green spaces (SPUGs), people preferred Social, and Social had a positive impact on stress restoration [28], because sociality is an indispensable demand for people [62]. In addition, social contacts can reduce the feelings of loneliness and increase perceived social support, and become the intermediary of green space to promote health [63]. This study also found the positive impact of Social on the stress restoration of forest park. Therefore, the influence of Social on stress restoration needs further research before more reliable conclusions can be drawn.

The restorative effect of Prospect can be explained from an evolutionary perspective. Human beings originated from grasslands with broad vision [21,64]. Hence, the Prospect-Refuge Theory (PRT) [53] believes that places with broad vision are conducive to discovering dangers and are more suitable for human survival, so people prefer open environments. Previous studies confirmed this view [65,66]. Similarly, study on children [67] and adolescents [33] demonstrated significant influence of Prospect on stress restoration.

In addition, this research did not find that Nature had a significant impact on stress restoration, which is inconsistent with the results of many studies [22,27,68]. The reason may be that for modern people who are used to living in cities, natural places may be considered desolate, which will reduce the sense of security [26].

Another result of this research was the interactions of LS and eight PSDs which had no significant effects on stress restoration, so there was no significant difference in the effect of the eight PSDs on the stress restoration between different stress groups. Therefore, although level of stress affects people’s preference for different dimensions [28,69], such preference does not affect the stress restoration. However, different types of research sites were selected and different scales were used to measure stress restoration in this study from previous research. Consequently, the influence of level of stress on the relationship between PSDs and stress restoration needs to be further studied.

### 4.3. Individual Characteristics Affecting Stress Restoration in Forest Park

This research showed that gender, age and visit frequency had significant impacts on the stress restoration of forest park, while visit duration also had an impact on the stress restoration but was insignificant. The effects of gender and age on stress restoration had been proven [33,70]. The assessment of women to stress restoration was significantly higher than men, which may be due to the higher perception of Nature, Refuge and Serene. Refuge and Serene were the two dimensions that had the most significant influences on stress restoration. However, studies of children and adolescents found that boys had higher perceived restoration than girls [70,71]. This study also found that in forest park, the evaluation of young people to stress restoration was lower than other age groups. This may be because the forest park’s landscape and environment were not up to the expectations of young people [33]. Nevertheless, Deng et al. [47] found no difference in stress restoration between genders and ages. Moreover, preference for the type of environment had been associated with stress recovery in many studies [50,51,72], which is different from the results of this study. This suggests that further studies are needed to clarify whether gender, age and preference for environment have impacts on the stress restoration of different types of green space.

As it has been proved before, there was a positive connection between frequency or duration people stay in nature areas and restoration from stress [73]. In nature dose framework proposed recently, frequency and duration of exposure to nature being beneficial for people’s health was emphasized again [44], which was further supported by our results. Researchers believed that the nature dose was a mediator in the health benefits people receive from nature [74], so visit frequency and visit duration were the key factors affecting the stress restoration. Therefore, in future research on the stress restoration of forest park, the frequency and duration of the visit to the research site should be considered.

### 4.4. Implications, Limitations and Further Study

According to our study, forest park has the stress restorative potential, so it needs more attention from researchers, administrators and the public. Among the measures to improve the management of forest park, improving Serene should be considered the most. Besides, due to the low perception of Social in forest park, forest park’s manager can consider appropriately improving Social by adding artificial facilities. At the same time, the Refuge of forest park also should be improved to enhance the stress restoration. For visitors, forest park has higher Prospect compared with other urban green spaces, thus it can be a good choice to enhance health condition. Additionally, it is necessary to conduct a survey about young people’s expectations of the forest environment because young people had a lower assessment of the stress restoration of forest park. The visit frequency and visit duration are usually affected by distance or commuting time. Therefore, the distance from major residential areas and accessibility of urban green space should be considered in future urban planning [75].

This study is not without limitations which should be addressed in the future. Firstly, this study was conducted in mountain-type forest parks in Beijing, and further research needs to be carried out in other types of forest parks, such as river and lake-type forest park, grassland-type forest park and waterfall-type forest park and so on [76]. Secondly, this study focuses on the relationship between perceived quality of forest park and stress restoration, rather than the impact of environmental types on stress restoration, which is also an important factor affecting the relationship [77]. Consequently, future studies on the relationship between PSDs and stress restoration should consider the differences caused by different environmental types. Thirdly, the perceived characteristics of forest park are the focus of this study. Future research can combine the objective characteristics with the perceived characteristics of restorative environment to conduct research [78].

## 5. Conclusions

This study took forest park as the research site to explore how PSDs are associated with stress restoration in Beijing. We obtained the following conclusions: (I) the perceived degree of PSDs in forest park from strong to weak was Serene, Space, Nature, Rich in species, Prospect, Refuge, Social and Culture, which varied with visitors’ gender, age, level of stress, visit frequency, activity intensity, visit duration and commuting time; (II) in PSDs, Refuge, Serene, Social and Prospect had significantly positive effects on the stress restoration of forest park; (III) there was no significant difference in the effect of the eight PSDs on the stress restoration between different stress groups; (IV) visitors’ gender, age, visit frequency and visit duration also affected the stress restoration of forest park. This research provides references for managers to improve the health benefits of forest park for visitors. It also enriches the research on PSDs and stress restoration as well as their relationship, providing a basis for future research in this content.

## Figures and Tables

**Table 1:** Questions on visiting characteristics of forest park and items.

Questions	1	2	3	4	5
visit purpose	exercising	relaxing	being with families or friends	observing nature	
visit frequency	very few	few	mediate	much	very much
activity intensity	very low	low	mediate	high	very high
visit duration	less than one hour	1–2 h	2–3 h	3–4 h	more than four hours
commuting time	less than one hour	1–2 h	2–3 h	3–4 h	more than four hours
number of companions	zero	one	two	three	four or more

**Table 2:** Perceived sensory dimensions (PSDs) and corresponding variables.

Perceived Sensory Dimensions (PSDs)	Variables
Nature	There is a nature quality.
	There is a wild and untouched quality.
	There are free growing lawns.
	One is able to spend time in the forest park without coming into contact with too many people.
Culture	The forest park is decorated with statues.
	The forest park has the characteristics of a city park.
	The forest park has different water features, like ponds, canals, etc.
Prospect	The lawns are cut.
	It is possible to have a prospect, vistas over the surroundings.
Social	It is possible to shop in market stalls, kiosks, etc.
	There are plenty of people and movements in the forest park.
	There is access to restrooms.
	There are tables and benches.
Space	The forest park has lots of trees.
	The forest park is experienced as spacious and free.
	It is possible to find areas not crossed by roads and paths.
	It is possible to find places where a company of several persons can gather.
Rich in species	One can detect several animals, like birds, insects, etc.
	The forest park consists of natural plant and animal populations.
	There are many native plants to study.
Refuge	It feels safe spending time in the forest park.
	The forest park contains many bushes.
	There is play equipment, like swings, slides, etc.
	It is possible to watch other people being active, playing, practicing sports, etc.
Serene	The forest park is silent and calm.
	There are no mopeds.
	The area is clean and well maintained.
	There is no traffic noise from the surroundings.

**Table 3:** The questionnaires issued in each forest park.

	Mangshan National Forest Park (MS)	Jiufeng National Forest Park (JF)	Xishan National Forest Park (XS)
The total number of the questionnaires	153	161	176
Number of valid questionnaires	136	145	151
Questionnaire effectiveness	89%	90%	86%

**Table 4:** The individual characteristics of the respondents in research sites.

		Mangshan National Forest Park (MS)	Jiufeng National Forest Park (JF)	Xishan National Forest Park (XS)	Percentage
Gender	Men	79	81	76	54.6%
	Women	57	64	75	45.4%
Age	<13	0	11	4	3.5%
	13–17	3	4	5	2.8%
	18–25	17	10	13	9.2%
	26–40	88	80	91	60.0%
	>40	27	41	38	24.5%
Prefer	Yes	36	44	47	29.4%
	No	100	101	104	70.6%
The level of stress	Average visitors	87	77	76	55.6%
	Stressed visitors	49	68	75	44.4%
	Sum	136	145	151	100.0%

**Table 5:** The factor load matrix of SRRS after rotation.

Variables	Common Factor Load			
	1	2	3	4
I feel grouchy—good natured	0.192	0.192	**0.878**	0.041
I feel anxious—relaxed	0.119	0.126	**0.915**	0.004
My breathing is getting faster	0.254	−0.001	0.075	**0.848**
My hands are sweating	−0.033	0.135	−0.028	**0.897**
I am interested in the present scene	**0.874**	0.303	0.195	0.137
I feel attentive to the present scene	**0.852**	0.364	0.177	0.109
I would like to visit here more often	0.286	**0.876**	0.152	0.092
I would like to stay here longer	0.328	**0.843**	0.210	0.069

Note: Bold is the maximum load of the variable on the common factor.

**Table 6:** The results of independent sample *t*-test results with gender, Prefer and LS as grouped variables and the eight PSDs as test variables.

Dimensions	Gender		Prefer		The Level of Stress	
	*t*	Mean Difference	*t*	Mean Difference	*t*	Mean Difference
Nature	−2.395 *	−0.171	−0.703	−0.055	1.936	0.138
Culture	−0.715	−0.065	0.588	0.058	2.000 *	0.181
Prospect	−0.921	−0.072	1.142	0.096	0.094	0.007
Social	−0.864	−0.068	1.513	0.129	0.853	0.067
Space	−0.648	−0.047	0.37	0.029	−0.205	−0.015
Rich in species	−1.91	−0.177	1.068	0.107	−0.365	−0.034
Refuge	−2.411 *	−0.180	0.273	0.022	−0.028	−0.002
Serene	−3.253 **	−0.233	−0.005	−0.000	−0.591	−0.042

Note: * *p* < 0.05, ** *p* < 0.01. Prefer = prefer forest park for outdoor activity or not; LS = level of stress in the last month.

**Table 7:** The results of ANOVA with age and VCs as independent variables and the PSDs as dependent variables.

		Nature	Culture	Prospect	Social	Space	Rich in Species	Refuge	Serene
Age	Sum of Squares	1.960	4.475	3.113	3.479	7.919	16.075	2.002	0.23
	Df	4	4	4	4	4	4	4	4
	Mean Square	0.490	1.119	0.778	0.870	1.980	4.019	0.501	0.058
	F	0.903	1.272	1.219	1.341	3.639 **	4.586 **	0.838	0.104
VP	Sum of Squares	0.180	0.036	1.546	1.195	0.075	2.876	2.323	0.429
	Df	3	3	3	3	3	3	3	3
	Mean Square	0.060	0.012	0.515	0.398	0.025	0.959	0.774	0.143
	F	0.110	0.014	0.812	0.611	0.045	1.078	1.324	0.266
VF	Sum of Squares	4.407	7.314	4.704	3.895	3.522	8.081	9.747	3.078
	Df	4	4	4	4	4	4	4	4
	Mean Square	1.102	1.829	1.176	0.974	0.880	2.020	2.437	0.770
	F	2.111	2.142	1.899	1.565	1.602	2.267	4.363 **	1.428
AI	Sum of Squares	14.755	5.320	2.211	0.273	4.098	5.137	1.000	0.713
	Df	4	4	4	4	4	4	4	4
	Mean Square	3.689	1.330	0.553	0.068	1.024	1.284	0.250	0.178
	F	7.347 **	1.52	0.877	0.105	1.858	1.434	0.425	0.332
VD	Sum of Squares	6.629	1.640	5.085	1.963	3.952	4.574	6.213	7.003
	Df	4	4	4	4	4	4	4	4
	Mean Square	1.657	0.410	1.271	0.491	0.988	1.143	1.553	1.751
	F	3.110 *	0.467	2.042	0.751	1.797	1.277	2.643 *	3.343 *
CT	Sum of Squares	2.253	2.798	8.008	2.755	3.907	0.895	2.623	1.198
	Df	4	4	4	4	4	4	4	4
	Mean Square	0.563	0.699	2.002	0.689	0.977	0.224	0.656	0.299
	F	1.061	0.794	3.247 *	1.064	1.769	0.246	1.118	0.557
NC	Sum of Squares	2.096	6.264	4.389	4.663	1.767	2.599	2.382	1.161
	Df	4	4	4	4	4	4	4	4
	Mean Square	0.524	1.566	1.097	1.166	0.442	0.650	0.596	0.290
	F	0.967	1.791	1.72	1.805	0.790	0.725	0.998	0.543

Note: * *p* < 0.05, ** *p* < 0.01; VCs = visiting characteristics to forest park; VP = visit purpose; VF = visit frequency; AI = activity intensity; VD = visit duration; CT = commuting time; NC = number of companions.

**Table 8:** Effects of PSDs on stress restoration.

Parameter	Estimation	Standard Error	df	*t*	F	Sig.
Intercept	2.169	0.124	259.371	17.433	303.901	0.000
Nature	0.044	0.037	389.739	1.178	1.388	0.239
Culture	−0.031	0.032	406.435	−0.962	0.926	0.336
Prospect	0.077	0.038	422.998	2.045 *	4.181 *	0.042
Social	0.126	0.040	384.400	3.138 **	9.844 **	0.002
Space	−0.008	0.046	422.467	−0.165	0.027	0.869
Rich in species	0.042	0.032	386.746	1.302	1.696	0.194
Refuge	0.143	0.041	32.986	3.489 **	12.175 **	0.001
Serene	0.127	0.038	422.978	3.361 **	11.296 **	0.001

Note: * *p* < 0.05, ** *p* < 0.01.

**Table 9:** The results of ANOVA with age and visiting characteristics as independent variables and the stress restoration index as dependent variable.

Factor	Sum of Squares	df	The Mean Square	F
Age	3.472	4	0.868	2.767 *
VP	1.238	3	0.413	1.294
VF	11.800	4	2.950	9.975 **
AI	0.385	4	0.096	0.298
VD	2.756	4	0.689	2.162
CT	1.614	4	0.404	1.260
NC	1.182	4	0.296	0.916

Note: * *p* < 0.05, ** *p* < 0.01; VP = visit purpose; VF = visit frequency; AI = activity intensity; VD = visit duration; CT = commuting time; NC = number of companions.

**Table 10:** Post hoc test results with age as independent variable and stress restoration index as dependent variable.

Age (I)	Age (J)	Mean Difference (I–J)	Standard Error	Sig.
18–25	<13	−0.341 *	0.170	0.045
	13–17	−0.453 *	0.184	0.014
	26–40	−0.259 **	0.095	0.007
	>40	−0.307 **	0.104	0.003

Note: * *p* < 0.05, ** *p* < 0.01.

**Table 11:** Post hoc test results with VF as independent variable and stress restoration index as dependent variable.

VF (I)	VF (J)	Mean Difference (I–J)	Standard Error	Sig.
very few	few	−0.074	0.073	0.316
	moderately	−0.387 **	0.070	0.000
	much	−0.336 **	0.104	0.001
	very much	−0.519 *	0.209	0.013
few	very few	0.074	0.073	0.316
	moderately	−0.314 **	0.085	0.000
	much	−0.262 *	0.115	0.023
	very much	−0.446 *	0.215	0.039

Note: * *p* < 0.05, ** *p* < 0.01; VF = visit frequency.

## Data Availability

No publicly archived datasets, analyzed or generated, were used in this study.
